# A possible role of astrocytes in contextual memory retrieval: An analysis obtained using a quantitative framework

**DOI:** 10.3389/fncom.2013.00145

**Published:** 2013-11-05

**Authors:** Shivendra Tewari, Vladimir Parpura

**Affiliations:** ^1^Biotechnology and Bioengineering Center, Medical College of WisconsinMilwaukee, WI, USA; ^2^Department of Physiology, Medical College of WisconsinMilwaukee, WI, USA; ^3^Department of Neurobiology, University of Alabama at BirminghamBirmingham, AL, USA; ^4^Department of Biotechnology, University of RijekaRijeka, Croatia

**Keywords:** hippocampus, CA3 region, CA1 region, contextual memory, astrocytes, computational biology

## Abstract

The hippocampus is central to our understanding of memory formation and retrieval. Its CA1 region is known for encoding contextual memory. Here, using a computational approach, which embeds existing physiological data, we propose a particular role of astrocytes in contextual memory retrieval. We provide a quantitative framework under which the astrocyte modulates the firing of a context-associated CA1 pyramidal neurons, resulting in a prominent tuning of neurons to a delta rhythm. Using the very framework, we further studied astrocytic function in the modulation of neuronal firing under pathological conditions, i.e., during astrocytic induction of epileptiform discharge in CA1 pyramidal neurons. Thus, we provide a quantitative framework that would aid understanding of the Schaffer collateral-CA1 tripartite synapse in health and disease.

## Introduction

One of the most intriguing properties of the brain is to learn and form new memories. Memories are essential for guiding effective actions, for understanding the words we read, for recognizing the objects we see, etc. It is well established that retrieval of a particular memory is facilitated when the context at the time of memory retrieval is similar to that of the initial learning experience (Maren and Holt, 2000). This phenomenon, termed as context-dependent memory, also provides an explanation as to why memory recall is impaired when people, place or things are experienced “out of context.” The process by which the context is embedded into learning of information, and can later facilitate memory retrieval is known as contextual memory retrieval (Maren and Holt, 2000). *Pavlovian* conditioning, i.e., classical conditioning, is an exemplar form of contextual fear memory learning and retrieval (Maren, 2001).

Ever since memory deficits, initially characterized in patients PB and FC (Milner and Penfield, 1955) and later HM (Scoville and Milner, 1957), were associated with hippocampus damage, it has become clear that the hippocampal region plays a crucial role in the acquisition of new semantic and episodic memory (Andersen et al., 2007); but see (Corkin, 2002). Apart from storing memories, the appropriate retrieval of stored memories is also of paramount importance for maintaining relationships, achieving personal and professional goals, and carrying out even simple day to day activities. The critical role of the hippocampus in memory retrieval has gone largely uncontested and has been supported by various studies including lesion studies (Holt and Maren, 1999), unit studies (Paller and McCarthy, 2002), as well as functional neuroimaging (Eldridge et al., 2000).

Lee and Kesner (2004) reported a sub–region specific contribution of the hippocampus in memory acquisition and retrieval. Interpretation of the result suggests that the CA1 region is uniquely associated with a reliable retrieval of the contextual memory. Such a role for the CA1 region in contextual memory retrieval has also been highlighted in several studies (Hall et al., 2001; Khuchua et al., 2003; Murchison et al., 2011). Previous work suggests that the firing synchrony in neighboring CA1 pyramidal neurons might be a putative mechanism of contextual memory retrieval in the CA1 region (Hall et al., 2001; Siekmeier et al., 2007; Takahashi and Sakurai, 2009). However, data are accumulating suggesting that the coordination between neurons and astrocytes may play a role in information processing and memory retrieval.

Fellin et al. (2004) demonstrated that the activation of Ca^2+^ elevation in astrocytes, by various stimuli, produces slow inward currents (SICs) in 2–12 CA1 pyramidal neurons, synchronously. They found that repetitive stimulation of astrocytes leads to synchronous firing of the same CA1 pyramidal neurons, mainly due to the activation of neuronal NR1/NR2B subunits of the extra–synaptic N–Methyl-D–aspartate receptors (eNMDARs). It should be noted that NMDAR NR2B subtype plays a crucial role in encoding and retrieval of contextual fear memory (Zhao et al., 2005; Zhang et al., 2008; Zhuo, 2009); but see Nakazawa et al. (2004). Thus, it appears that the interaction/signaling between neurons and astrocytes plays a role in information processing and memory retrieval.

Astrocytes in the hippocampus have numerous highly branched fine processes that extend to distances up to 100 μm and enwrap asymmetrical (mainly excitatory/glutamatergic) synapses of a relatively small group of neurons (Fellin et al., 2006); of note, individual astrocytic domains do not overlap (Bushong et al., 2002, 2004; Ogata and Kosaka, 2002; Nedergaard et al., 2003; Oberheim et al., 2009). Thus, strategically, astrocytes are ideally positioned to sample electrical activity patterns of the Schaffer collateral input from CA3 pyramidal neurons and retrieve their associated context in neighboring CA1 pyramidal neurons. During the retrieval, the firing pattern of the pyramidal neurons, in the CA3–CA1 region, is quite similar to that recorded during memory formation (Small et al., 2001). As astrocytes play an important role in short-term and long-term potentiation of individual synapses (Martin et al., 2007; Henneberger et al., 2010), it is possible that they also contribute to various aspects of memory. We propose that astrocytes facilitate the memory storage and retrieval by sensing encoding/retrieval patterns of neurons that reside within individual astrocytic domains and, in turn, astrocytes signal the associated CA1 pyramidal neurons.

Based on these recent experimental observations, we developed a computational framework/model, where an astrocyte modulates synchrony among CA1 pyramidal neurons within a domain of 100 μm in diameter, which qualitatively reproduces the biological experimental findings (Fellin et al., 2004). Power spectral density analysis of the computed local field potentials indicates a shift in neuronal firing rhythm from broad-frequency range in the absence of astrocytic gliotransmission to a delta-dominant rhythm when gliotransmission is turned on. As delta rhythm has been implicated in cognitive domain (Knyazev, 2012) and consolidation of declarative memories (Ruch et al., 2012), this might be a putative mechanism of memory retrieval tuned by astrocytes, which will require further experimental verification in biological preparations. Apart from a possible role in memory retrieval, using the computational model, we propose that astrocytes may lead to epileptiform discharges in CA1 pyramidal neurons, as has been previously demonstrated in biological preparations (Kang et al., 2005; Tian et al., 2005). Such pathological discharges occuring under computational conditions warrant an increase in eNMDAR plasmalemmal activity that could arise from an overexpression of such receptors and/or positive modulation of their conductance (Omkumar et al., 1996; Liao et al., 2001).

## Materials and methods

### Overview of the model architecture

**Figure 1:**
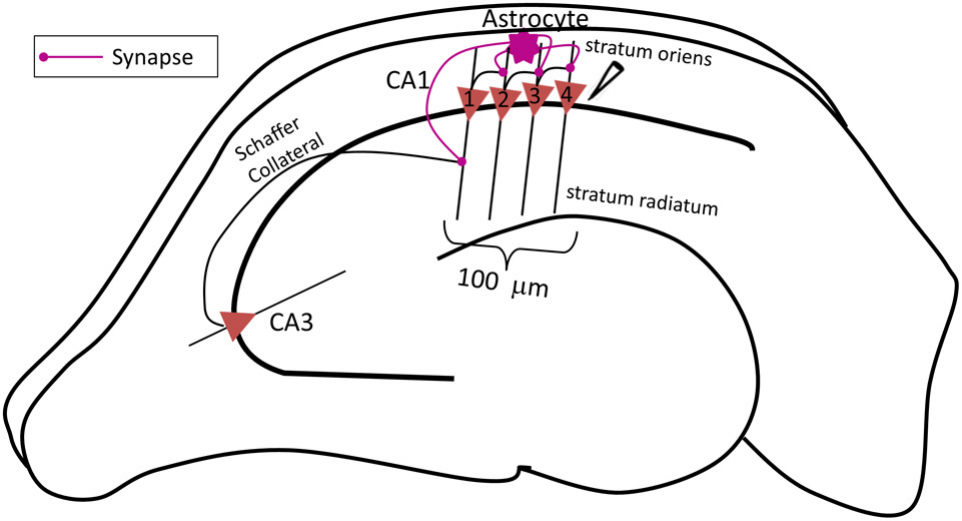
**Schematic diagram of the proposed network model of contextual memory retrieval within the hippocampus where an astrocyte modulates synchronous firing of a pack of four CA1 pyramidal neurons during context-dependent memory retrieval.** The pack of neurons is aligned within 100 μm of the stratum pyramidale along the input, i.e., Schaffer collateral (SC) axons of the CA3 pyramidal neuron, which makes synapses with the spines of the proximal apical dendrites of CA1 pyramidal cells in the stratum radiatum. The field potential recording electrode is placed next to the CA1 neuron 4, at the most distal end to the input of this pack. The fine processes of the astrocyte, allied with the neuronal pack and located in the stratum oriens, listen to the synaptic transmission which takes place in the stratum radiatum, at the synapse made by the CA3 pyramidal neuron onto the lead CA1 pyramidal neuron 1 (the utmost left, proximal to the input). The astrocyte integrates received information and relays the signal to the following CA1 pyramidal neurons within the pack (in this case the three remaining CA1 pyramidal neurons 2–4, distal to the input) associated with the context of the input signal from the CA3 pyramidal neuron concurrently, the astrocyte also potentiates the SC-CA1 synapse strengthening, the small network activity.

The complete network (shown in Figure 1) comprises of three major components: a CA3 pyramidal neuron, four CA1 pyramidal neurons and an astrocyte in the *stratum oriens* (s.o.). A CA3 pyramidal neuron is computationally described by Traub's branching dendrite model of the CA3 pyramidal neuron comprising of 21 compartments (Traub et al., 1994). Each of the four CA1 pyramidal neurons is described by the 19-compartment CA1 pyramidal neuron model (Traub et al., 1991), which was modified to incorporate four additional compartments accounting for the axon initial segment (IS), axon proper, and two spines (placed at the 6^th^ and 12^th^ compartment of the original CA1 pyramidal neuron model) to yield a 23-compartment CA1 pyramidal neuron model. Axon and spine compartments were added to form CA1–CA1 synapses in the s.o. at the basal dendrites. The dendritic spine model utilized here is from Tewari and Majumdar (Tewari and Majumdar, 2012a), but was modified to include synaptic NMDARs and eNMDARs. The astrocyte model is also adopted from Tewari and Majumdar (Tewari and Majumdar, 2012a,b), and it accounts for gliotransmitter (glutamate) release/astrocyte-dependent SICs through the activation of NR2B-containing receptors on the CA1 neurons. It is assumed that the CA3 pyramidal neuron makes 25 synapses with the CA1 neuron 1 (Figure 1). The number of synapses at the CA1–CA1 synapses is assumed to be 20. These numbers were obtained by a hit and trial approach, and reflect the smallest numbers possible to have a successful signal transduction of the proposed network. All associated model equations and parameters are provided in the supplementary materials.

As shown in Figure 1, the CA3 neuron forms synapses with the CA1 neuron 1, at the spines in the *stratum radiatum* (s.r.). To model glutamate release from the CA3 and CA1 neurons, we use a continuous function that transforms axon voltage into glutamate concentration in the cleft (Destexhe et al., 1994):

(1) $$[g]=\frac{g_{\text{max}}}{1+\exp\text{​}\left[-\left(V_{\text{axon}}-V_{\text{p}}\right)/K_{\text{p}}\right]},$$

where *g*_max_ is the maximum concentration of glutamate in the synaptic cleft, *V*_axon_ is the membrane potential in the axon compartment of the glutamate releasing neuron, *K*_p_ gives the steepness, and *V*_p_ is the axon voltage at which glutamate concentration is half of the maximum (see Table 1). The glutamate released from CA3 neuron is sensed by the astrocyte in the s.o. (Perea and Araque, 2005) leading to astrocytic Ca^2+^ elevation. Simultaneously, glutamate released from the CA3 neuron leads to an inward current in the CA1 neuron 1. The SICs evoked by the astrocyte through eNMDARs are:

(2) $$I_{\text{SIC}}=g_{\text{NR2B}}\cdot h\cdot v_{\text{s}},$$

where *g*_NR2B_ is the conductance through NR1/NR2B-containing receptors, *v*_s_ is the spine membrane potential and *h* is the gating variable of eNMDAR that depends on the astrocyte as described by the following equation:

(3) $$\frac{dh}{dt}=\frac{\sigma }{\tau_{1}}-\frac{h}{\tau_{2}}.$$

**Table 1:** **List of parameters involved at the synapses**.

**Symbol**	**Value**	**Reference**
*g*_max_	3.5–4 mM	Danbolt, 2001
*V*_p_	72 mV^†^	Destexhe et al., 1994
*K*_p_	5 mV	Destexhe et al., 1994
*g*_NR2B_	0.6 nS	Gasparini et al., 2004
τ_1_	92.3 ms	Fellin et al., 2004
τ_2_	568.5 ms	Fellin et al., 2004

† *This value is reported with respect to the resting membrane potential*.

The above relation is regulated by astrocytic Ca^2+^ by σ, which has a value of 0.4 (representing the maximum open probability of eNMDAR) whenever astrocytic Ca^2+^ goes above 200 nM (Parpura and Haydon, 2000; Nadkarni and Jung, 2003; Tewari and Majumdar, 2012b), otherwise set to zero. The time constants τ_1_ and τ_2_ govern the rise-time and decay-time of the eNMDAR currents, and are based on the experimental observation (Fellin et al., 2004); parameter values are listed in Table 1.

A given CA1 neuron forms synapses with the other CA1 neurons in the basal dendrite region (Andersen et al., 2007); in the proposed network model, the synapses are formed at the 6^th^ compartment of the CA1 neuron model. The sequence in which the CA1 neurons make synapses is as follows: 1→2, 2→3, 3→4 (Figure 1, left to right); the CA1 neuron 4 is not connected to any other neuron. The astrocyte senses the signal (glutamate) released by the CA3 neuron and potentiates the above CA1 neuron synapses by releasing gliotransmitter (glutamate) in the vicinity of the eNMDARs on the spines, which leads to synchronized firing of the CA1 neurons. The astrocyte releases glutamate from glutamate secretory vesicles utilizing Ca^2+^-dependent exocytosis as soon as astrocytic Ca^2+^ concentration exceeds the threshold Ca^2+^ concentration for triggering secretion [see (Tewari and Majumdar, 2012a) for details].

### Computer simulations

The proposed network model invoked a total of 1247 ordinary differential equations which were solved using a quasi-constant step sized backward-difference numerical solver implemented in MATLAB (The MathWorks, Natick, MA). The system of ordinary differential equations was solved for a duration of 50 s (model time) which took approximately 8 h (real time) to compute.

### Local field potential calculations

The somata of the CA1 neurons were spaced 25 μm apart. The recording electrode was placed 25 μm away from the soma of the CA1 neuron 4 (see Figure 1). The local field potential was calculated using line source approximation (Gold et al., 2006). Based on the ionic currents generated from the network simulation (in the presence and absence of the astrocyte), trans-membrane currents across each compartment were calculated for each of the CA1 neurons. Please note that the contribution from IS and axon compartments was neglected because myelinated axon and nodes of Ranvier make no contribution to the local field potential (Gold et al., 2006). Afterwards, linear source approximation was used to compute local potentials at the recording electrode using a custom-written MATLAB program. Mathematically, field potential is calculated using the following expression:

(4) $$\Phi (t)=\sum_{\forall \text{c}}\frac{\rho_{\text{c}}I_{\text{c}}}{4\pi r_{\text{c}}},$$

where *I*_c_ is the trans-membrane current across compartment c, *r*_c_ is the distance of compartment c from the recording electrode, and ρ_c_ is the extracellular resistivity at compartment c. The value of ρ_c_ for soma and proximal apical dendritic compartments is 643 Ω cm (Lopez-Aguado et al., 2001), while for the basal and distal apical dendritic compartments it is 260 and 287 Ω cm, respectively (Lopez-Aguado et al., 2001).

## Results

### Network simulations

**Figure 2:**
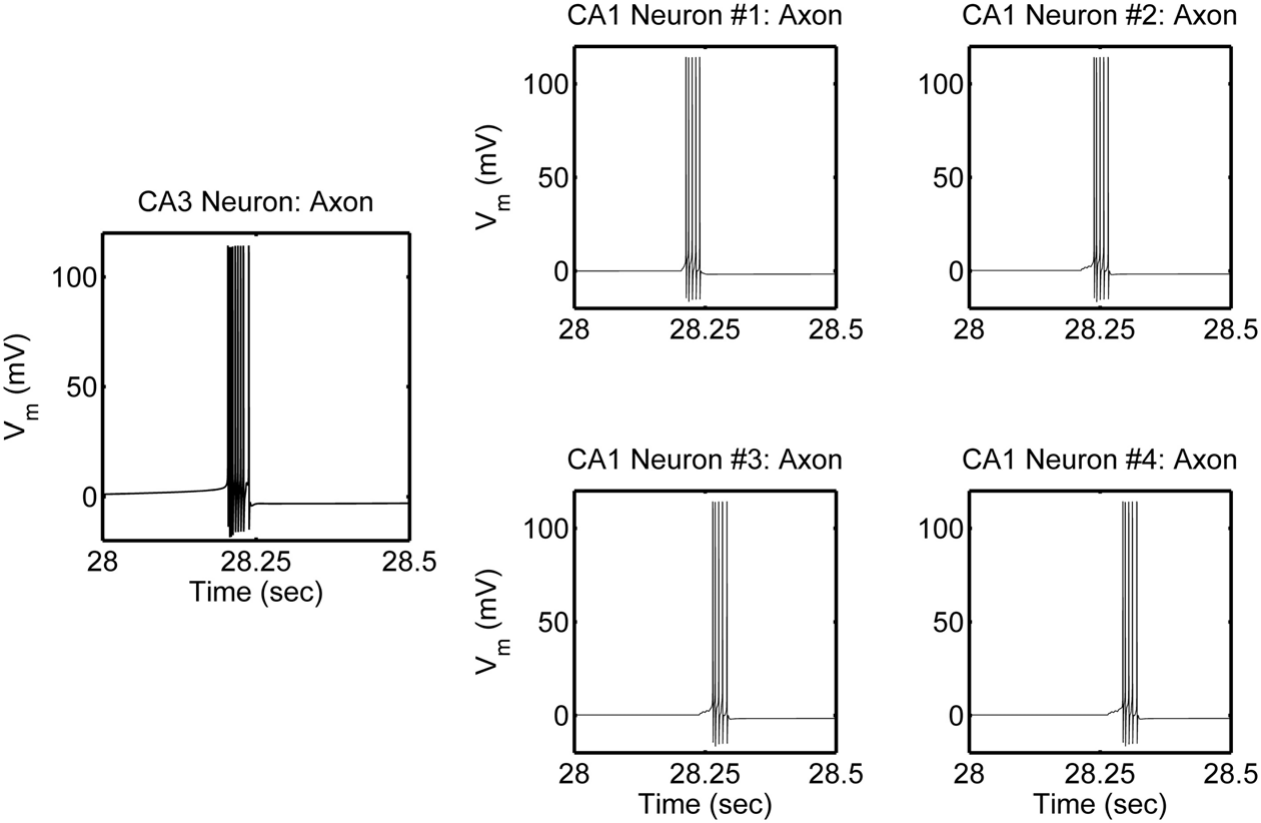
**Model simulations of the CA3–CA1 pyramidal neuron network (shown in Figure 1), in the absence of s.o. astrocytic activity, showing action potential generation in the axon compartment of the CA3 and CA1 pyramidal neuron models.** The CA3 neuron was given a constant current of 0.6 nA, in the soma compartment, which evokes a high-frequency burst of ~100 Hz and of various durations, which repeats itself at ~0.66 Hz. There were corresponding responses in the post-synaptic CA1 neurons.

**Figure 3:**
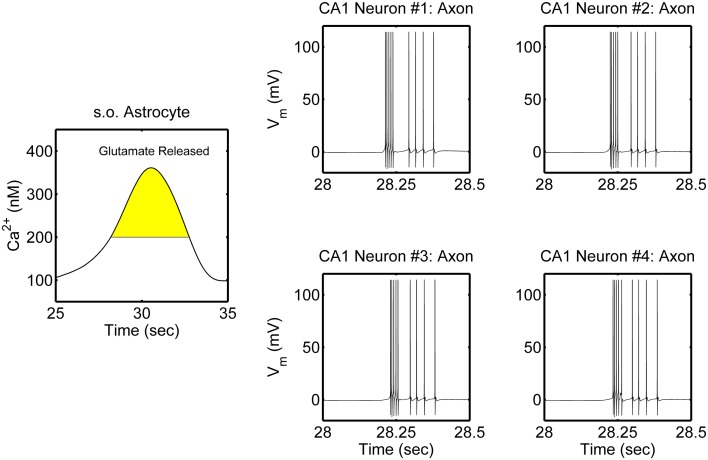
**Model simulations of the CA3–CA1 pyramidal neuron network (shown in Figure 1), in the presence of s.o. astrocytic activity i.e., gliotransmission.** The CA3 neuron was stimulated again in the soma with an input current of 0.6 nA (therefore the CA3 neuron activity is not shown). Representative astrocytic Ca^2+^ cycle and its effect on the CA1 neurons are shown. The shaded area of the astrocytic Ca^2+^ indicates the time during which SICs are induced in the basal dendrites of the CA1 neurons 2–4 and the apical dendrites of the CA1 neuron 1.

**Figure 4:**
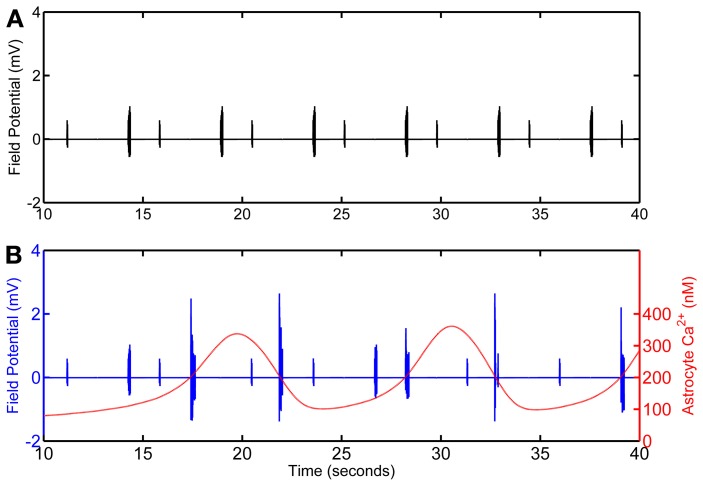
**Calculated field potential based on model simulations of the network in the absence (A) and in the presence (B) of the astrocyte.** The simulation omits the first 10 s of simulation as, in the model, astrocytic Ca^2+^ oscillations begin after 10 s of the CA3 neuronal activity.

The network is simulated in the absence and in the presence of astrocytic activity, i.e., a feedback to neurons in respect to synaptic transmission. Figure 2 shows representative action potential (AP) bursts in the axon compartment of the CA3 neuron that lead to subsequent firing in the CA1 neurons 1–4, with a slight delay, as expected from the flow of the input signal in the CA1 region. The network was then simulated again, while incorporating the astrocytic response to neuronal synaptic transition, i.e., gliotransmission, that leads to an induction of SICs in the CA1 neurons. We show a representative astrocytic response and its associated effects on the firing of the CA1 neurons (Figure 3). It is apparent by comparing the two different simulations (compare Figures 2 and 3; and Figures 4A and 4B) that the astrocyte makes the CA1 neurons to fire in synchrony, and in a different pattern. Note that the astrocyte induces SIC twice (data not shown; this effect is captured by the gating variable *h* in Equation 3) while the astrocytic Ca^2+^ concentration is above the threshold for triggering exocytotic release of glutamate (see the shaded area in Figure 3).

### Local field potentials

**Figure 5:**
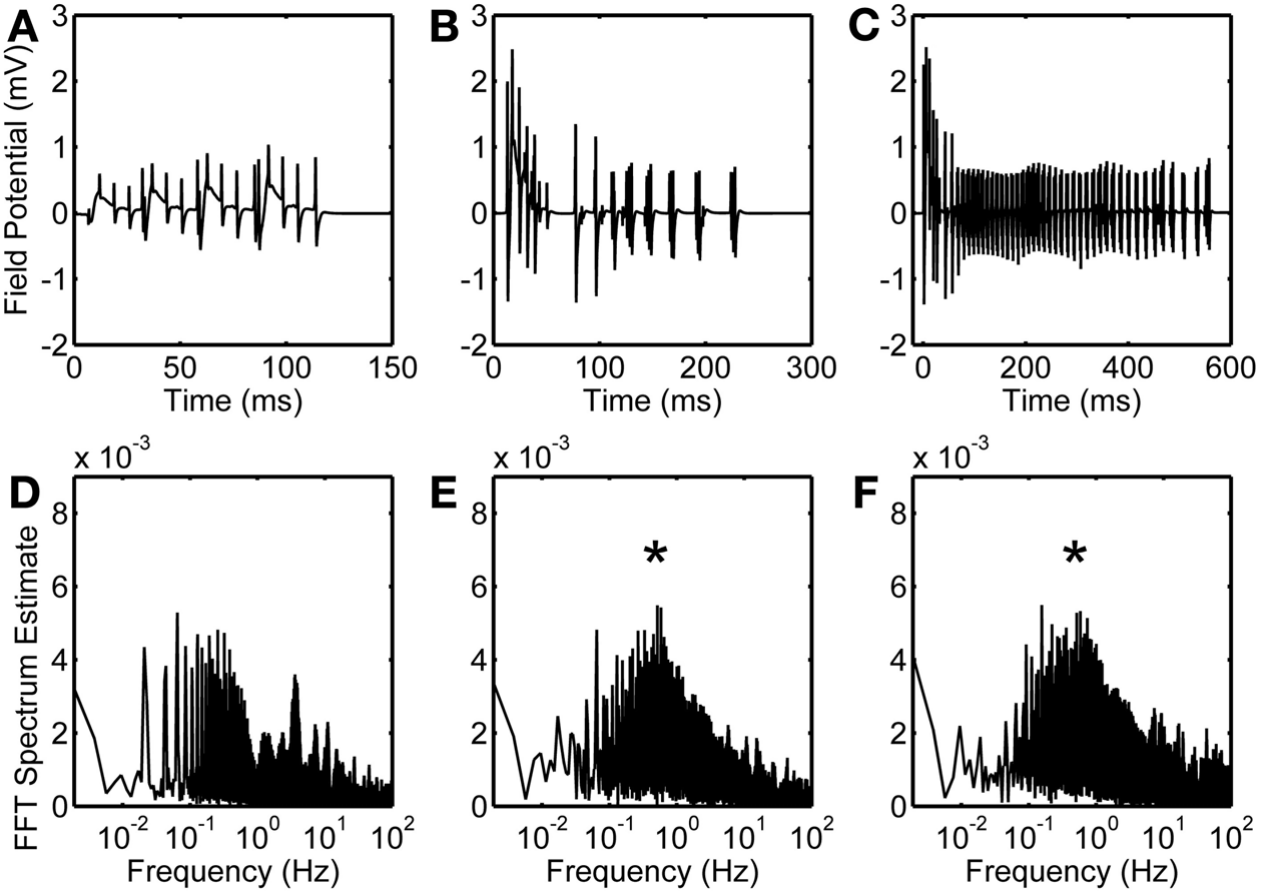
**Astrocytic contribution to field potential recordings.** Duration of evoked field potentials in the absence of astrocyte **(A)**, in the presence of astrocyte activity **(B)**, and in the presence of astrocytic activity along with an enhanced eNMDAR conductance in CA1 neurons **(C)**. Corresponding FFT spectra are shown in **(D,E**, and **F)**, respectively. Note that FFT spectra are calculated based on complete simulated data of 50 s. Asterisks denote the effect of astrocyte-induced SICs.

Local field potential (LFP) calculation at the point of interest is based on linear source approximation in the absence and presence of the astrocyte. It is apparent from the peaks of LFP that the CA1 neurons fire synchronously under the astrocyte-dependent pathway (Figures 4 and 5). Hence, after the addition of the astrocyte-induced SICs, the CA1 neuron pack starts firing at a different rate, which is dependent on astrocyte Ca^2+^ oscillations (Figure 4). The LFP duration is much shorter in the absence of astrocytic activity (Figure 5A) compared to the LFP duration in the presence of an active astrocyte (Figure 5B); the input signal, from the CA3 neuron, was same for both pathways. To assess a possible difference in the dominant frequency between the two pathways, we used Fast Fourier Transform (FFT) spectrum analysis (Figures 5D,E). Based on this analysis, the astrocyte-independent pathway has wide-spread activity with multiple peaks, a pattern likely resulting due to the differences in the firing rate of each CA1 neuron within the pack; the main proportion of the activity forms an envelope, around 4–11 Hz, lacking any typical rhythm (see Figure 5D). The FFT analysis of the astrocyte-dependent pathway shows a shift towards lower frequency with sharpened tuning, resulting in an occurrence of a delta rhythm (Figure 5E, asterisk), possibly due to synchronous firing of the CA1 neurons in tune with the astrocyte gliotransmission.

### Epileptiform simulations

NMDARs are known to play a role in the initiation and propagation of epileptiform discharges (Auzmendi et al., 2009). To study the possible effect of glutamatergic gliotransmission on the eNMDARs, as demonstrated in biological preparations [e.g., (Fellin et al., 2004)], we simulated the astrocyte-dependent pathway after doubling the eNMDAR conductance in the CA1 neurons (Table 1). This computational alteration emulates a biological condition of an increased presence of plasmalemmal eNMDARs (due to an overexpression/increased trafficking) and/or flux through an unaltered number of channels. As a control, we simulated the same network under conditions incapacitating gliotransmission of the s.o. astrocyte, caused by clamping astrocytic Ca^2+^ at 100 nM, which is below the threshold for glutamate release. The simulation data suggest that the presence of the astrocyte lacking gliotransmission does not affect the firing of the CA1 neurons overexpressing eNMDARs upon their stimulation by the CA3 neuron (compare Figure S1 and Figure 2). However, the astrocytic activity/gliotransmission may induce epileptiform discharges in the CA1 neurons via an increased eNMDAR activity, as evidenced by the grossly exaggerated duration of LFPs (Figure 5C). FFT spectrum analysis of the epileptiform activity further indicates that the astrocyte activity tunes the CA1 neurons to fire in a delta rhythm, while other surrounding frequencies appeared suppressed (Figure 5F, asterisk).

## Discussion

Herein we presented a biophysically detailed mathematical framework of the CA3/Schaffer collateral-CA1 pyramidal neuron signal transduction that is based on experimental reports suggesting a possible role of astrocytes in contextual memory retrieval by synchronizing CA1 pyramidal neuronal network and achieving a delta rhythm. We were interested in studying a small neuronal network for two reasons: (i) a small network model can be kept biophysically detailed, and (ii) to overcome the experimental limitations in biological preparations (i.e., a lack of the spatial resolution to record field potentials from such a small network, as opposed to recording from a much larger area in reality). We did analysis of the simulated local field potentials generated in the absence and in the presence of astrocytic activity. The results suggest that astrocytic gliotransmission potentiates the synapses through eNMDARs, synchronizes CA1 neurons and modulates their overall firing rate. Frequency domain analysis of the simulated LFP suggests that astrocytic Ca^2+^ signaling shifts the neuronal activity in the CA1 region from a broad spectrum frequency lacking any typical rhythm towards a delta frequency. Interestingly, a recent study (Grover et al., 2009) described the induction of long-term potentiation in the CA1 region due to a burst stimulation over a broad frequency range centered around delta frequency, albeit the underlying mechanism was not investigated. Thus, our *in silico* model offers a possible computational explanation to this delta frequency and it should be additionally explored in biological preparations.

Delta rhythm is a low frequency brain activity which is implicated in cognitive domain such as attention, salience detection, and subliminal perception (Knyazev, 2012). Moreover, it is known that sensory cues innervate the CA1 pyramidal neurons associated with the context. Under such circumstances repetition of an item stimulus will recover the contextual state, in the entorhinal cortex, in response to firing of the associated pyramidal cells in the CA1 region. In our circuitry model, we observe that astrocytic input, which makes CA1 pyramidal neurons to fire in a synchronous manner, eliminates group firing activity at other frequencies except delta, suggesting a possible role of astrocytes in memory retrieval. From a functional point of view, this finding proposes a cognitive role of astrocytes where an environmental cue demands attention, e.g., delta rhythm generation in visual cortex depends on the activity to which attention is paid (Lakatos et al., 2008). The model suggests that similar activity of delta rhythm generation might exist in the CA1 region of hippocampus during events of contextual memory retrieval.

NR2B subunit overexpression enhances chronic pain in mice (Zhuo, 2009). Additionally, NR2B-containing NMDARs are linked to cell death and seizures (Ding et al., 2007). To test the functional consequence of NR2B overexpression on CA1 neuronal activity, we increased the conductance of NR2B-containing NMDARs which is equivalent to an insertion of eNMDARs in the plasmalemma or enhanced unitary conductance of the eNMDARs. Such a condition simulates an increased access of astrocytic transmission to the NR2B-containing NMDARs. The results suggest that enhanced conductance of NR2B-containing NMDARs leads to a high-frequency oscillation of field potentials. The duration of these field potentials is ~600 ms which is equal to the duration of SIC via NR2B-containing NMDARs. This activity could be a molecular correlate of seizures seen in the hippocampus, which are sensitive to NR2B subunit antagonists (Kolarova et al., 2003; Butler et al., 2010).

In our present work, we have not adjusted our model for micro-anatomical restructuring at the tripartite synapse, which would almost inevitably occur due to the dynamics at astrocytic filopodia/perisynaptic processes (Hirrlinger et al., 2004; Lavialle et al., 2011). Such restructuring could lead to changes in the proximity of neuronal and astrocytic release and/or input sites. Future dynamic modeling to simulate micro-anatomical arrangements would be needed to computationally address the role that astrocytic structural plasticity may play in tuning neuronal firing rhythm.

### Conflict of interest statement

The authors declare that the research was conducted in the absence of any commercial or financial relationships that could be construed as a potential conflict of interest.
