# Supplementary material for: A possible role of astrocytes in contextual memory retrieval: An analysis obtained using a quantitative framework
Source: Front Comput Neurosci. 2013 Nov 5;7:145. doi: 10.3389/fncom.2013.00145 (PMC3817599; doi:10.3389/fncom.2013.00145)
Supplement: Supplementary file 1 [file DataSheet1.PDF]

Supplementary materials to

"A possible role of astrocytes in contextual memory retrieval: An analysis obtained using a quantitative framework"

by

Shivendra Tewari and Vladimir Parpura

Model Equations

CA1 Axon Initial Segment & Axon Compartment

The CA3 pyramidal neuron model is Traub's branching dendrite model (Traub et al., 1994), therefore the underlying model equations are not shown. We made modifications to the Traub's CA1 pyramidal neuron model (Traub et al., 1991) by including four additional compartments: the axon initial segment (IS), axon proper (axon) and two spines. The below mentioned discrete form of the cable equation is used to connect the compartments together:

$$C_{\mathrm{k}} \frac{dV_{\mathrm{k}}}{dt} = \sum_{\mathrm{l}} \gamma_{\mathrm{l,k}} \left(V_{\mathrm{l}} - V_{\mathrm{k}} \right) - I_{\mathrm{ionic,k}}. \tag{1}$$

Here l represents the compartment connected to compartment k, C is the membrane capacitance, V_{k} is the trans-membrane voltage, γ is the coupling conductance between the connected compartments, and the sum is over all the compartments connected to k. $I_{\mathrm{ionic,k}}$ is the total ionic membrane current across the compartment k. Coupling conductance is calculated based on the following expression:

$$\gamma_{\mathrm{k,l}} = 2 / \left(\rho_{\mathrm{k}}^{-1} + \rho_{\mathrm{l}}^{-1} \right); \; \rho_{\mathrm{x}} = R_{\mathrm{i}} L_{\mathrm{x}} / \pi r_{\mathrm{x}}^2,$$

where ρ is the internal resistance of a compartment (x, either k or 1); R_i is the internal resistivity of the compartment x, and r_x and L_x are the radius and length of the compartment x, respectively. The internal resistivity of the IS and axon compartments is assumed to be equal and is equal to 0.1 kΩ cm (Traub et al., 1994). The membrane capacitance for each of the compartments is taken to be 0.75 μF cm^{-2}. All other parameters are listed in Table S1.

The membrane ionic current for the IS and axon compartments is given by the following equation:

$$I_{\mathrm{ionic,k}} = g_{\mathrm{L,k}} V_{\mathrm{k}} + \bar{g}_{\mathrm{Na,k}} m_{\mathrm{k}}^3 h_{\mathrm{k}} \left(V_{\mathrm{k}} - V_{\mathrm{Na}} \right) + \bar{g}_{\mathrm{K(DR),k}} n^4 \left(V_{\mathrm{k}} - V_{\mathrm{K}} \right). \tag{2}$$

$g_{\mathrm{L,k}}$ is the leak conductance, V_{k} is the local trans-membrane potential with respect to resting membrane potential. $\bar{g}$ denotes the maximum conductance of the voltage-gated channel (Na, sodium; K(DR), potassium-delayed rectifier) for the compartment k. The maximum conductance can be determined from conductance densities and compartment membrane areas (see Table S1). V_{Na} and V_{K} are the equilibrium potentials for respective ions, also with respect to resting membrane potential. *m*, *h*, *n* are dimensionless gating variables that govern the kinetics of a particular ion channel. These variables are of Hodgkin-Huxley type formulism and assumed to have the same kinetics as the IS and axon of the CA3 pyramidal neuron (Traub et al., 1994).

Table S1. CA1 IS-axon parameters; from (Traub et al., 1994)

**Conductance Densities**			
**Parameter**	**Value**		
g_{Na}	500 mS cm^{-2}		
$g_{\mathrm{K(DR)}}$	250 mS cm^{-2}		
g_{L}	1 mS cm^{-2}		
**Reversal Potentials***			
**Ion**	**Value**		
K^+	-15 mV		
Na^+	115 mV		
**Compartment Sizes**			
	Radius (μm)	Length (μm)	Area (μm^2)
Initial segment	2	75	942
Axon proper	0.5	75	236

* Note: with respect to the resting membrane potential of -70 mV; thus, E_{K^+} = -85mV and E_{Na^+} = +45 mV

CA1 Spines

The basic spine model is almost the same as previously described (Tewari and Majumdar, 2012), with the sole modification to include synaptic NMDARs and extra-synaptic NMDARs (eNMDARs). The current through eNMDARs is described in the main text. The synaptic NMDAR current is given by the following equation:

$$I_{\mathrm{NMDAR}} = g_{\mathrm{NMDAR}} B(v_{\mathrm{s}}) r v_{\mathrm{s}}. \tag{3}$$

Here g_{NMDAR} is the maximal conductance through NMDAR, v_{s} is spine-head membrane potential. $B(v_{\mathrm{s}})$ is the function that governs the voltage-dependent Mg^{2+} block of NMDAR given by:

$$\frac{1}{1+\exp(-0.062 v_{\mathrm{s}})\dfrac{[Mg^{2+}]_{\mathrm{syn}}}{3.57}},$$

where $[Mg^{2+}]_{\mathrm{syn}}$ refers to Mg^{2+} concentration in the synaptic cleft. r is non-dimensional variable representing the fraction of open NMDARs. It is described by the following first-order differential equation:

$$\frac{dr}{dt} = \alpha g_{\mathrm{syn}} (1-r) - \beta r, \tag{4}$$

α and β represent the rate at which NMDARs open and close. The rate at which NMDARs open is dependent on the concentration of glutamate in the synaptic cleft (g_{syn}), which comes from Equation 1 of the main text. The spine-neck resistance is assumed to be 95.4 MΩ (Koch, 1999). All other parameter values are listed in Table S2.

Table S2. CA1 Spine-head parameters; from (Destexhe et al., 1994)

**Parameter**	**Description**	**Value**
g_{NMDAR}	Maximum NMDAR conductance	0.01 nS
α	NMDAR forward rate constant	7.2×10^4 M^{-1} s^{-1}
β	NMDAR backward rate constant	6.6 s^{-1}
$[Mg^{2+}]_{\mathrm{syn}}$	Synaptic Mg^{2+} concentration	1 mM

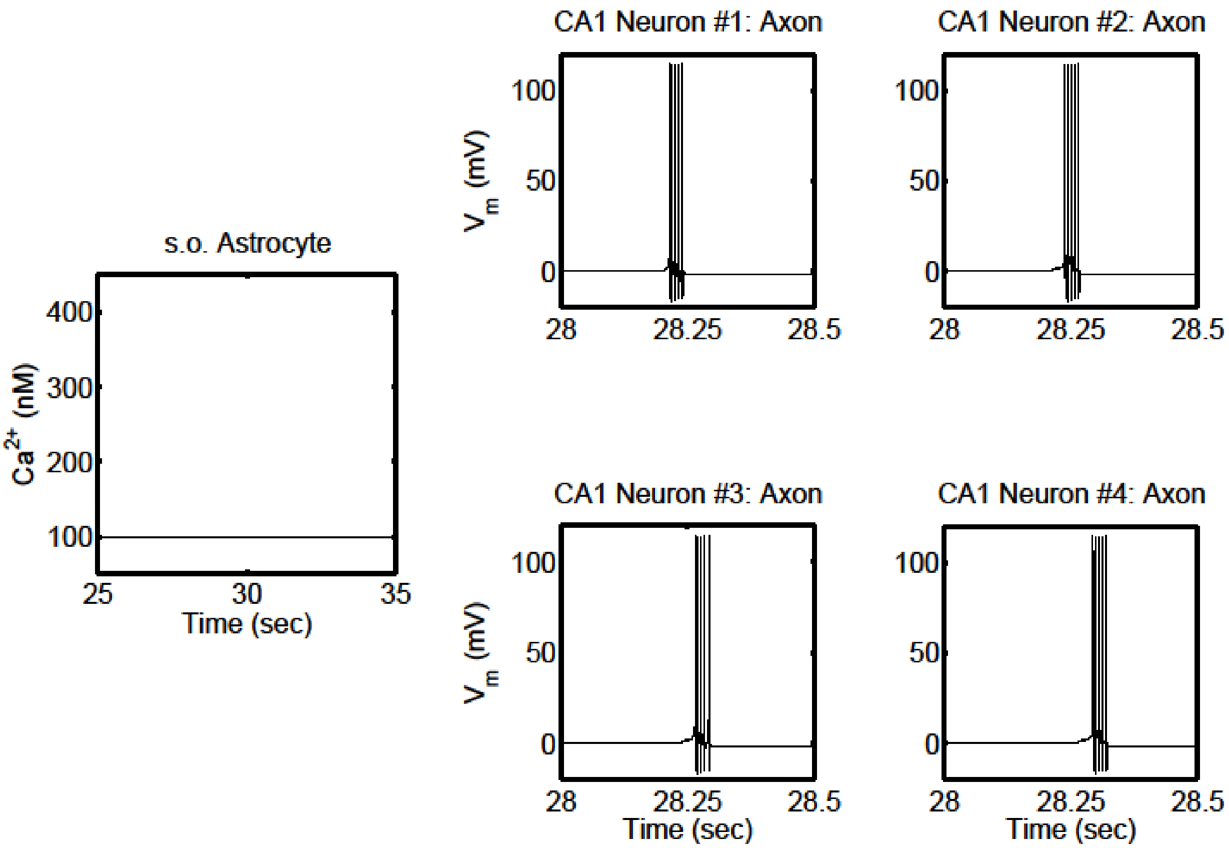

Figure S1. Model simulations of the CA3-CA1 pyramidal neuron network (shown in Figure 1) with an enhanced eNMDAR conductance in the CA1 neurons, and in the presence of the s.o. astrocyte whose intracellular Ca^{2+} is clamped (at 100 nM) below the threshold for glutamate release. The CA3 neuron was stimulated again in the soma with an input current of 0.6 nA (therefore the CA3 neuron activity is not shown). Presence of the astrocyte with incapacitated gliotransmission, owing to the Ca^{2+}clamp, does not affect the firing of the CA1 neurons (compare to Figure 2).

References:

Destexhe, A., Mainen, Z.F., and Sejnowski, T.J. (1994). Synthesis of models for excitable membranes, synaptic transmission and neuromodulation using a common kinetic formalism. *J Comput Neurosci* 1**,** 195-230.

Koch, C. (1999). *Biophysics of computation : information processing in single neurons.* New York: Oxford University Press.

Tewari, S.G., and Majumdar, K.K. (2012). A mathematical model of the tripartite synapse: astrocyte-induced synaptic plasticity. *J Biol Phys* 38**,** 465-496.

Traub, R.D., Jefferys, J.G., Miles, R., Whittington, M.A., and Toth, K. (1994). A branching dendritic model of a rodent CA3 pyramidal neurone. *J Physiol* 481 (Pt 1)**,** 79-95.

Traub, R.D., Wong, R.K., Miles, R., and Michelson, H. (1991). A model of a CA3 hippocampal pyramidal neuron incorporating voltage-clamp data on intrinsic conductances. *J Neurophysiol* 66**,** 635-650.
